# Pancreas-Specific Physical Examination Enabling Early Diagnosis of Pancreatic Injury in Children: A Case Report

**DOI:** 10.7759/cureus.81478

**Published:** 2025-03-30

**Authors:** Takateru Ihara, Osamu Nomura, Miki Ishikawa, Akihiro Shimotakahara, Naoki Shimojima, Takaaki Mori

**Affiliations:** 1 Department of Pediatrics, Hyogo Prefectural Amagasaki General Medical Center, Hyogo, JPN; 2 Department of Pediatric Emergency and Critical Care Medicine, Tokyo Metropolitan Children’s Medical Center, Tokyo, JPN; 3 Medical Education Development Center, Gifu University, Gifu, JPN; 4 Department of Surgery, Tokyo Metropolitan Children’s Medical Center, Tokyo, JPN; 5 Department of Emergency Medicine, KK Women's and Children's Hospital, Singapore, SGP

**Keywords:** intra-abdominal injury, isolated pancreatic trauma, pediatric, pediatric abdominal surgical emergency, physical diagnosis

## Abstract

Pediatric pancreatic injuries are rare but require early diagnosis. Ihara’s maneuver, a pancreas-specific palpation technique, is useful for the early diagnosis of pediatric pancreatic injuries. We herein report a case of pancreatic injury detected by Ihara’s maneuver. An early adolescent male patient presented with abdominal pain and vomiting following an abdominal contusion. His vital signs were normal. Ihara’s maneuver induced slight rebound tenderness in the left hypochondrium. Laboratory tests demonstrated elevated serum amylase and lipase. Contrast-enhanced computed tomography revealed a distal pancreatic injury with slight fluid retention. Magnetic resonance cholangiopancreatography and endoscopic retrograde cholangiopancreatography were required owing to worsening abdominal pain. A distal pancreatic transection with pancreatic ductal injury was diagnosed, and a partial pancreatic resection was performed. The patient was discharged 57 days after hospitalization. Ihara's maneuver, an anatomically specific palpation technique, is useful for the early detection of pancreatic injuries in pediatric patients.

## Introduction

Blunt pancreatic injuries are uncommon in children. A study of pediatric patients with an intra-abdominal injury found that 39%, 37%, 19%, and 7% of the cohort had an injury to the spleen, liver, kidneys, and pancreas, respectively [1]. Another study reported that pancreatic injuries accounted for only 0.7% of all pediatric abdominal traumas. Thus, pancreatic injuries are the fourth most common type of solid organ injury in pediatric blunt abdominal trauma cases and occur far less frequently than injuries to the spleen, liver, or kidneys [2]. When they do occur, they sometimes require surgery to treat the inflammation resulting from pancreatic juice leakage. Delayed diagnosis can lead to pseudocyst, recurrent pancreatitis, fistula, or abscess formation. However, while early diagnosis of pancreatic injuries is critical, it is also usually challenging. First, laboratory tests, including serum amylase and lipase, have limited sensitivity and specificity for pancreatic injuries [3]. Focused assessment with sonography in trauma scan is limited in its ability to detect pancreatic injuries, as it does not sufficiently cover the anatomical region of the pancreas. On the other hand, despite its effectiveness in evaluating intra-abdominal organ injuries, computed tomography (CT) exhibits insufficient sensitivity specifically for pancreatic injuries. Magnetic resonance cholangiopancreatography (MRCP) and endoscopic retrograde cholangiopancreatography (ERCP) can more accurately evaluate pancreatic injuries, including ductal injuries, but are highly invasive and require general anesthesia in children [4]. For these reasons, in patients with a history of trauma, a physical examination is critical to arriving at a clinical diagnosis by enabling pretest assessment for a pancreatic injury. The utility of Ihara’s maneuver, a pancreas-specific palpation technique, was previously reported for early diagnosis of pancreatitis and pancreatic tumors [5,6]. During this maneuver, the physician places both hands at the midpoint between the xiphoid process and umbilicus on the left midclavicular line and applies gentle, vertical pressure, gradually increasing it while moving the hands toward the spine to achieve deeper palpation. This maneuver allows for direct manual pressure on the pancreatic body and tail by displacing the stomach. We herein reported an early diagnosis of a pancreatic injury using this maneuver, which demonstrated its applicability not only to endogenous conditions such as pancreatitis but also to traumatic cases.

## Case presentation

**Figure 1:**
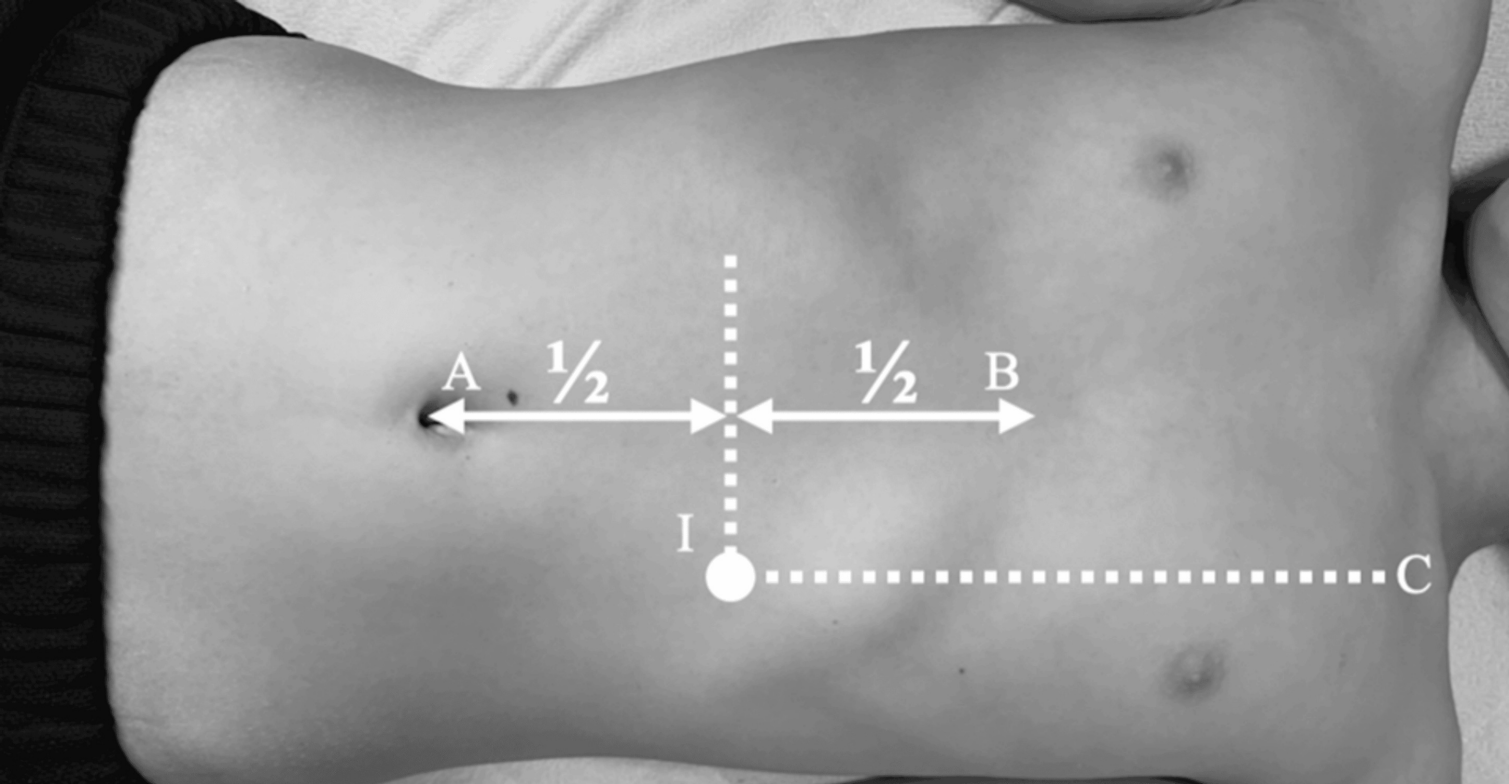
Anatomic benchmarks in Ihara’s maneuver. The pressure point in Ihara’s maneuver (I) is located midway between the umbilicus (A) and xiphoid process (B) on the left midclavicular line (C)

**Figure 2:**
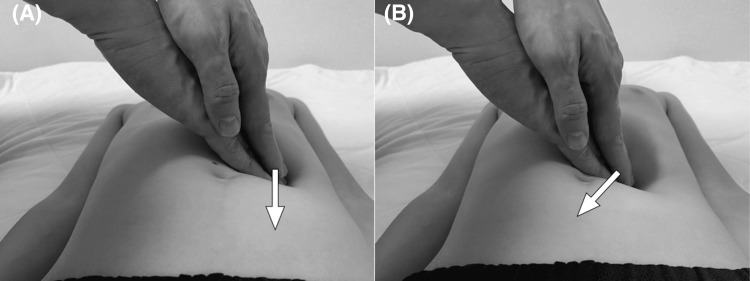
Performing Ihara’s maneuver. With the fingertips, apply slight pressure vertically on the point shown on the patient’s back (A), then increase the pressure while moving the fingertips toward the patient’s spine to achieve deeper palpation (B)

**Figure 3:**
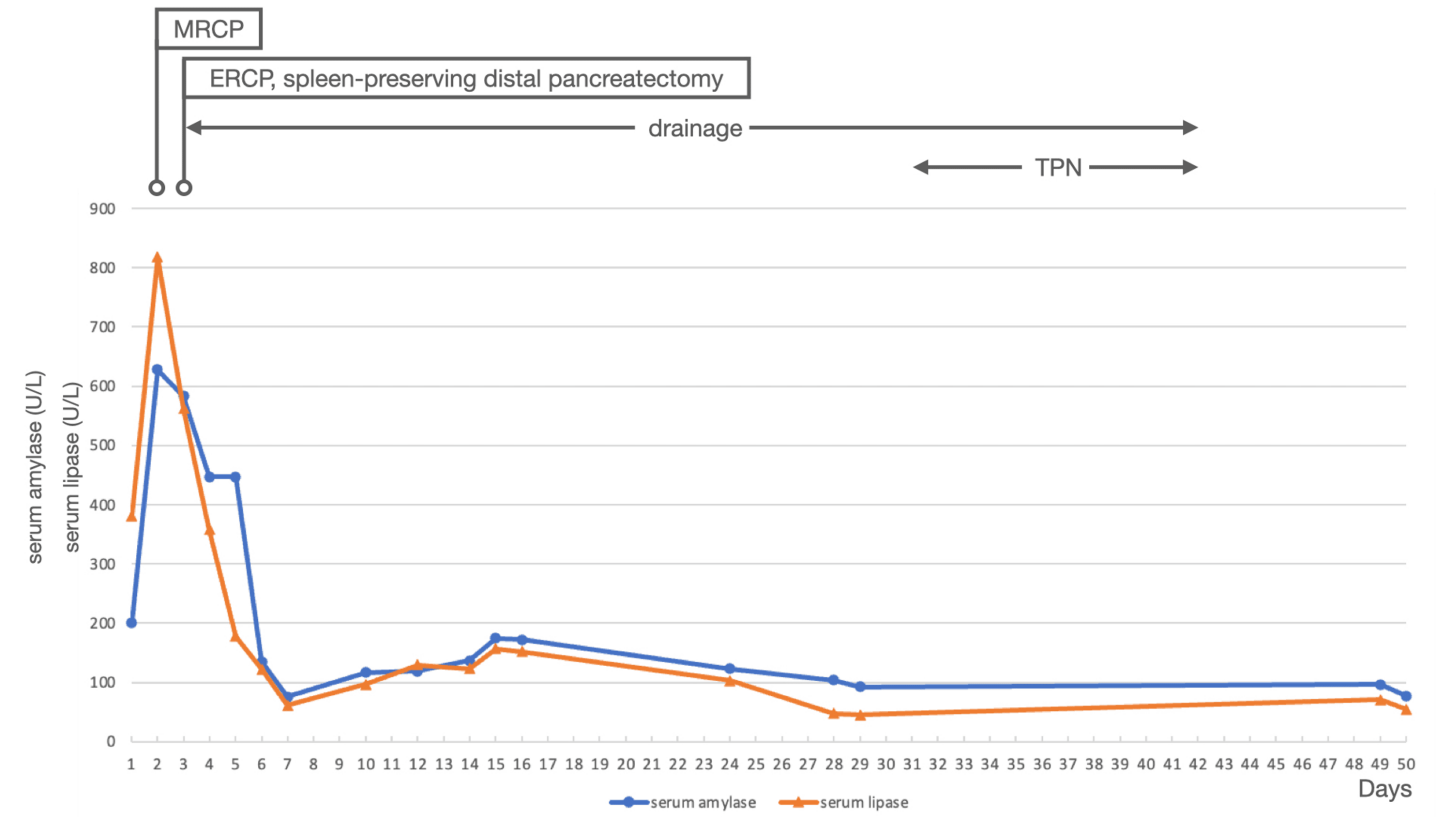
Changes in serum amylase and lipase after injury MRCP: magnetic resonance cholangiopancreatography; ERCP: endoscopic retrograde cholangiopancreatography; TPN: total parenteral nutrition

**Figure 4:**
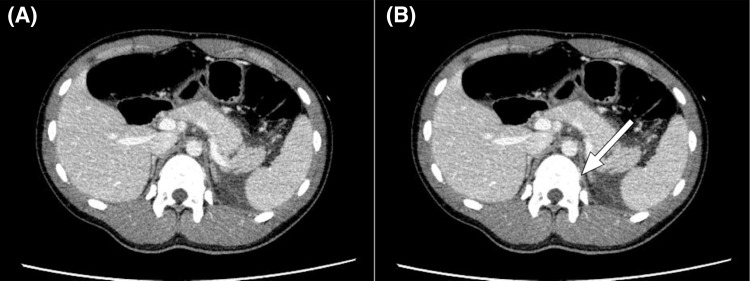
Abdominal CT scan of the patient showing a distal pancreatic transection with a small, peripancreatic fluid collection (American Association for Surgery of Trauma grade III) (A). CT found compression at the site of the injury corresponding to the area of compression between the vertebral body and the examiner’s hand during Ihara’s maneuver, as indicated by the arrow (B)

An otherwise healthy, 14-year-old male patient visited our emergency department with abdominal pain following an abdominal contusion sustained during a game of football when another player struck his upper abdomen. During the 90 minutes following the injury, the abdominal pain gradually worsened, and he experienced several episodes of vomiting. His pulse rate, blood pressure, respiratory rate, oxygen saturation, and body temperature were 74 bpm, 150/81 mmHg, 20 breaths/minute, 97% in room air, and 36.4℃, respectively. On physical examination, the abdomen was flat, soft, and without bruising. Ihara’s maneuver caused severe pain, and tenderness was not observed at any site other than those to which pressure was applied during the maneuver (Figures 1, 2). No other signs of trauma were observed in any other area. A complete blood count demonstrated elevated white cells (12.3 × 10⁹/L), normal hemoglobin (14.2 g/dL), and normal platelets (188 × 10⁹ / L). His electrolytes, urea, and creatinine were normal, but his lipase (381 U/L) and amylase (201 U/L) were elevated (Figure 3). Aspartate aminotransferase was also mildly elevated at 50 U/L, while C-reactive protein was normal at 0.06 mg/dL. A contrast-enhanced abdominal CT revealed a distal pancreatic transection with a small peripancreatic fluid collection (Figure 4A). The patient initially underwent conservative management, as contrast-enhanced CT at presentation revealed minimal peripancreatic fluid collection, and no worsening of laboratory or abdominal findings was observed. On postinjury day (PID) 1, MRCP was unable to visualize the main pancreatic duct at the injury site, leading to a diagnosis of main pancreatic duct disruption. On PID 2, ERCP demonstrated significant contrast extravasation, indicating severe main pancreatic duct injury. An endoscopic nasopancreatic drainage tube was placed, and the patient later underwent spleen-preserving distal pancreatectomy with the placement of a left subdiaphragmatic and pancreatic stump drain on the same day.

Although oral intake was initiated early postoperatively, a fever developed, necessitating temporary bowel rest and antibiotic therapy. On postoperative day (POD) 24, the patient experienced abdominal pain, prompting contrast-enhanced CT, which revealed an adhesive small bowel obstruction. Conservative management with bowel rest was initiated. The left subdiaphragmatic drain was removed on POD 26, and total parenteral nutrition via a peripherally inserted central catheter (PICC) was started on POD 31. The pancreatic stump drain and PICC were removed on POD 42. The patient gradually increased oral intake and was discharged 51 days after admission on POD 48 without complications.

## Discussion

Blunt pancreatic trauma accounts for only 0.6% of pediatric abdominal injuries [2]. Its diagnosis is often delayed because the symptoms and physical examination findings are nonspecific. Abdominal pain occurs in 72%-87% of patients but is not specific to pancreatic injuries [7,8]. The location of tenderness on physical examination is also nonspecific. Adelgais et al. reported epigastric tenderness in only 16.6% of patients [9]. Peritoneal irritation caused by a pancreatic injury was found to be less common in children than in adults, with only 37.5% of pediatric patients experiencing this symptom [8]. Persistent elevation of serum amylase and lipase is insufficient for diagnosis in the early period after an injury. Serum amylase and lipase levels in pancreatic injuries vary with the time elapsed since injury, and their sensitivity and specificity are not clearly defined. Mahajan et al. reported that a respective rise in the amylase and lipase values exceeding 250 and 100 international units had a sensitivity and specificity of 85% and 100%, respectively [3]. However, they found that in 71% of patients, serum amylase and lipase were not elevated within six hours of sustaining a pancreatic injury. Therefore, the authors concluded that these markers could not be used to exclude a pancreatic injury within six hours of the event. CT is the first-line imaging modality for diagnosing pancreatic injuries in hemodynamically stable patients with blunt trauma. Direct signs of pancreatic injury on CT include lacerations, focal enlargement, and hematomas. Contusions appear as hypoenhanced areas accompanied by peripancreatic edema, while lacerations appear as low-attenuation areas with a linear or branching pattern. Indirect signs include peripancreatic fat stranding and fluid collection. Studies of pancreatic injuries in adults have reported a sensitivity of 70%-95% for multidetector CT [10]. However, several studies of pediatric pancreatic injury found that 33%-53% of cases had normal CT findings but were laparoscopically diagnosed as a pancreatic injury [11]. MRCP and ERCP are useful for assessing for a main pancreatic duct injury but are more invasive and time-consuming, making them less suitable for patients in unstable condition. The most invasive yet definitive diagnostic approach is surgery, which should be considered in such patients.

In the present case, a pancreatic injury was suspected when Ihara's maneuver, which involves a specific palpation technique, induced tenderness. In this technique, the pancreas is compressed between the physician’s hand and the vertebral body while the stomach is entirely unaffected. Initially, CT found compression at the site of the injury corresponding to the area of compression between the vertebral body and the examiner’s hand during Ihara’s maneuver, as indicated by the arrow in Figure 4B. The tenderness experienced by the patient may have been caused by pancreatic fluid leakage, which occurs during the early phase of a pancreatic injury. Previous reports have described cases of acute pancreatitis and pancreatic tumor diagnosed with Ihara’s maneuver [5,6]. In these studies, Ihara’s maneuver was instrumental in detecting pancreatic inflammation at an early stage of pancreatitis. In solid, pseudopapillary neoplasms of the pancreas with intratumoral bleeding, the maneuver induced localized tenderness by increasing internal pressure. Applying indirect pressure to the pancreas makes certain pancreatic pathologies, such as lesions with elevated internal pressure, more sensitive to palpation, thereby aiding in their identification. Similarly, Ihara’s maneuver can be used to diagnose a pancreatic injury by compressing the site of the injury.

The indications for this palpation technique may be limited. First, Ihara’s maneuver is a palpation technique for assessing the pancreas, and therefore, it cannot distinguish a pancreatic injury from pancreatitis or a pancreatic tumor. The patient’s clinical history should be considered when interpreting the findings. Additionally, this method may have less diagnostic value in cases where deep palpation is difficult to perform, such as in patients with severe obesity. Furthermore, generalized pain may be difficult to localize specifically to the pancreas, and if the patient has altered consciousness status, performing the technique may be challenging. The maneuver relies on compressing the body and tail of the pancreas, but assessing injuries to the pancreatic head is anatomically more difficult. However, 70% of pancreatic injuries occur in the body and tail [1,2]. The extent to which the utility of this maneuver varies with the severity of the pancreatic injury and the duration from the infliction of the trauma remains unclear. However, as it is less invasive and repeatable, it may be used not only for early assessments but also to monitor patients whose test findings are unable to confirm the presence of a pancreatic injury. Further studies are warranted to elucidate the diagnostic utility of Ihara’s maneuver under different clinical conditions.

In conclusion, pancreatic injuries in children are rare and sometimes misdiagnosed due to the nonspecificity of symptoms and physical examination findings and the insufficient sensitivity of abdominal CT. Ihara's maneuver can pinpoint pancreatic injuries by inducing tenderness in the organ using an anatomically specific compression technique. Therefore, this procedure is useful for the early diagnosis of pancreatic injuries.

## Conclusions

Despite the mild mechanism of injury, the pancreatic trauma in the present case was detected early using Ihara’s maneuver, an anatomically specific palpation technique. Pediatric pancreatic injuries are rare and often difficult to diagnose owing to the limited sensitivity of abdominal CT and serum amylase and lipase values. The addition of Ihara’s maneuver may aid the early detection of pancreatic injuries.
